# Designing a molecular magnetic button based on 4*d* and 5*d* transition-metal phthalocyanines

**DOI:** 10.1038/s41598-017-03920-5

**Published:** 2017-06-16

**Authors:** P. Ferriani, S. Heinze, V. Bellini

**Affiliations:** 1 Institute of Theoretical Physics and Astrophysics, University of Kiel, D-24098 Kiel, Germany; 2 S3-Istituto di Nanoscienze-CNR, Via Campi 213/A, I-41125 Modena, Italy

## Abstract

The field of molecular spintronics exploits the properties of organic molecules possessing a magnetic moment, either native in the form of radicals or induced by the insertion of transition metal magnetic ions. To realize logic or storage molecular spin-tronics devices, molecules with stable different magnetic states should be deposited on a substrate, and switching between the states controllably achieved. By means of a first-principles calculations, we have devised a functional molecule exhibiting different magnetic states upon structural changes induced by current injection. We investigate the prototypical case of non-planar M-Phthalocyanine (MPc), where M is a transition-metal ion belonging to the 4*d* and 5*d* series. We find that for ZrPc and HfPc deposited on a graphene decorated Ni(111) substrate, two different structural conformations could be stabilized, for which the molecules attain different magnetic states depending on the position of the M ion *–* whether above the Pc or between the Pc and the substrate *–*, acting therefore as molecular magnetic button. Our work indicates an intuitive way to engineer a magnetic molecular switch with tailored properties, starting from the knowledge of the basic atomic properties of elements and surfaces.

## Introduction

One of the main research lines in magnetism is to embed the spin degrees of freedom into a molecular unit, taking advantage of the monodispersity of an ensemble of synthesized molecules with identical magnetic properties. The insertion of metal ions in an organic framework has been shown to be a successful route to design magnetic molecules with tailored properties^1, 2^, Concordantly, the interest in assembling spintronic devices by grafting molecular building blocks onto solid state scaffolds has been steadily growing in the last years^3, 4^.

A system with two stable magnetic states is necessary to perform logic operations and to store information in magnetic media. For this purpose the ability to control the magnetization of the system and switch it from one state to the other is also required. Several switching mechanisms have been demonstrated for metalorganic molecules adsorbed on surfaces^5^. A modification of the oxidation state of a molecule’s metal center, e.g. by controlled adsorption or desorption of ligands or doping of the substrate surface, has been found to effect a variation in its spin state or magnetic anisotropy^6–13^. High-spin to low-spin transitions have also been found to occur via temperature, pressure, strain, electric field and current variation in so-called spin-crossover molecules^14–19^.

Controlled switching of the spin state of individual molecules has been also achieved using a scanning tunneling microscope (STM) tip, with the added advantage that molecules can be addressed individually. For instance, the mechanical control of a single-molecule junction has been obtained by slowly approaching and retracting the tip thereby switching its magnetic and conductance properties^20–22^. Such methods are however of difficult integration in nanoscale devices. An easier control can be achieved using an electrical current or voltage. This route has been shown in STM experiments to be successful to induce a rotation of a Pc ligand in a TbPc_2_ molecule^23^ and the deformation of a tetracyanoethyelene molecule, resulting in creation and quenching of the magnetic moment^24^, and to activate a spin transition in a spin-crossover complex^25^.

**Figure 1:**
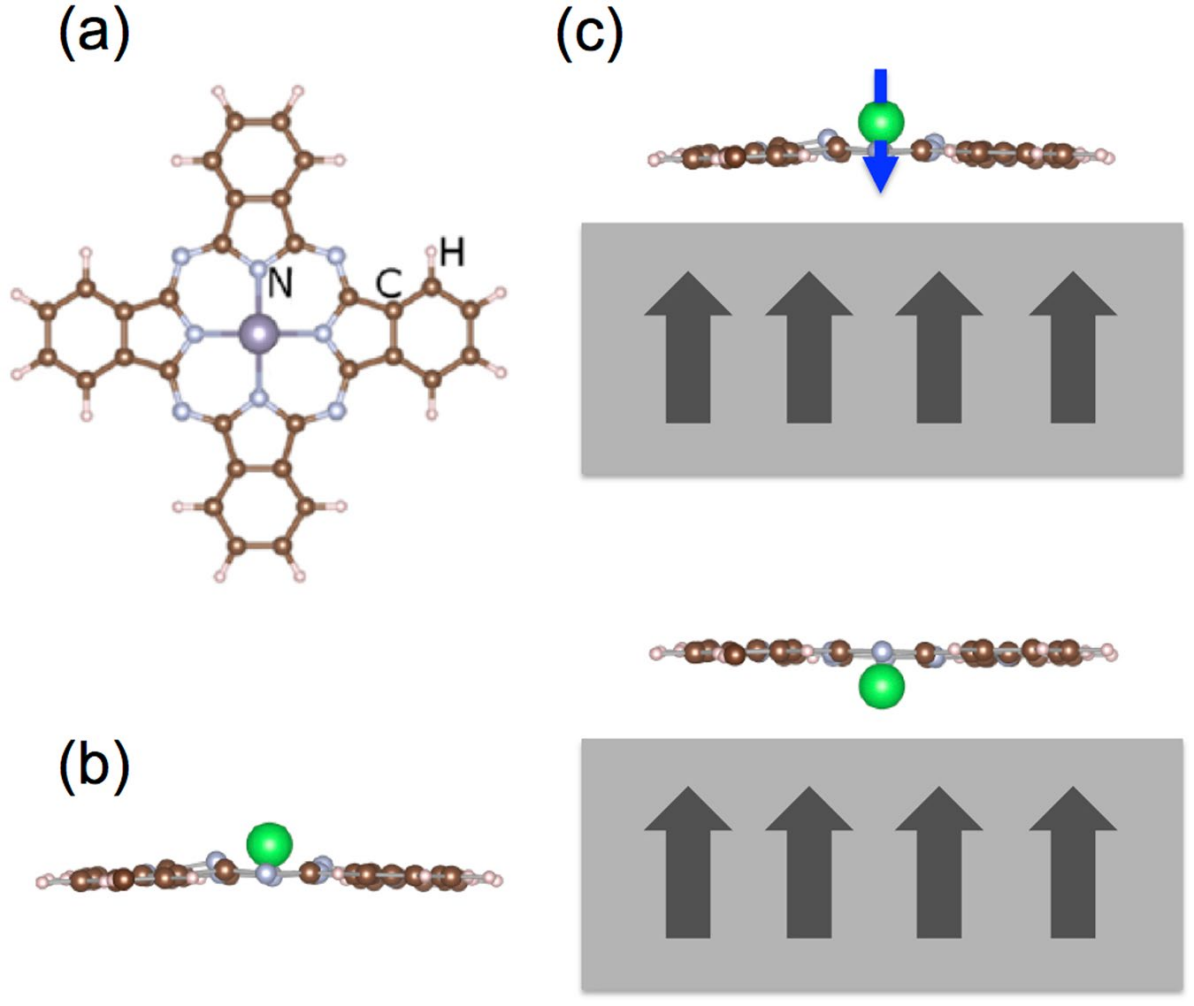
(**a**) Chemical structure of a M-Pc molecule. (**b**) Side view of the special type of M-Pc molecules exhibiting a shuttlecock shape, (**c**) Sketch of the concept of a molecular magnetic button: A M-Pc molecule is adsorbed on a magnetic substrate. Depending on the adsorption configuration, i.e. whether the metal ion is above the molecular plane or between the molecular plane and the substrate surface, the metal ion exhibits a magnetic moment or not.

Along this line, conformational changes via voltage pulses have been observed for the non-magnetic and non-planar SnPc molecule^26^. Due to the large size of the metal ion, such a molecule attains a shuttlecock shape with the metal ion protruding from the otherwise flat Pc organic framework (Fig. 1). SnPc can then acquire two conformations on a surface, either with the central metal atom pointing away from (MPc-*up*) or toward (MPc-*down*) the surface. Interestingly, upon locally charging the Sn ion by a current pulse induced by a STM tip, it is possible to push the ion through the Pc plane, as demonstrated for the case of the Ag(111) substrate^26^. This process is proved to be reversible when the SnPc molecules are adsorbed on other SnPc molecular layers^26^. Thus, SnPc can be regarded as the molecular equivalent of a two-state “button”, where in principle binary information can be stored.

Here we pursue further this idea, and we investigate whether such a functionality could be implemented in the presence of an additional degree of freedom, i.e. a magnetic moment, in such a way that the two different structural configurations correspond to different magnetic states and thus obtain a *magnetic button*. As a natural extension of the SnPc button, we consider the case of a single metal ion spin bound to a Pc molecular block, i.e. MPc, where M is a transition metal. In order to retain the bistable structural conformation the molecules are supported on a low interacting substrate. In addition, we are interested in the case where stabilization of the magnetic moment is achieved by the interaction with a magnetic substrate. As an ideal candidate, we identify the graphene decorated Ni(111) surface (G/Ni(111)), which has been shown to preserve to a large extent the properties of adsorbed molecules and atoms, simultaneously allowing sizable adsorbate-substrate magnetic interaction^27–31^. We found that ZrPc and HfPc deposited on a graphene layer on Ni(111) exhibit the *up* and *down* structural configuration similar to SnPc whereas the magnetic moment is retained only in the *up* state, i.e. ZrPc and HfPc are stable two-state magnetic systems. The transition barrier between such states is smaller than the one observed in the case of SnPc^26^, which suggests that also the ZrPc and HfPc molecules can be switched without detrimental effects. Our approach is theoretical, and we have employed state of the art density-functional theory methods, as implemented in the VASP simulation package^32, 33^ (further details on the calculations can be found in the Method section).

## Results

### The choice of the metal ion: a first selection from simplified calculations

To begin with, we show the criteria that we followed to pinpoint the MPc molecules exhibiting two stable magnetic and structural configurations. First, we verified the presence of a molecular spin and a non-planar structural conformation for the isolated molecule. In the case of 3*d* TM-Pcs, the metal ion, due to its small radius, is well accommodated inside the Pc macrocycle. This class of molecules has only one structural conformation and can thus be excluded from our quest. This is the case also for rare-earth ions, e.g. Tb, whose ionic radius is so large that the ion remains far away from the organic plane and the molecule is stabilized only if sandwiched with an additional Pc, leading to double- or multi-decker structures^34^. Good candidates to realize a bistable, switchable magnetic molecule are Pc molecules containing a 4*d* or 5*d* TM ion. In fact, several elements belonging to such rows of the periodic tables do not form double-decker structures and at the same time have an ionic radius that does not fit to the Pc cavity, leading to a structure similar to SnPc. Thus, we focus our study on such a class of molecules.

**Figure 2:**
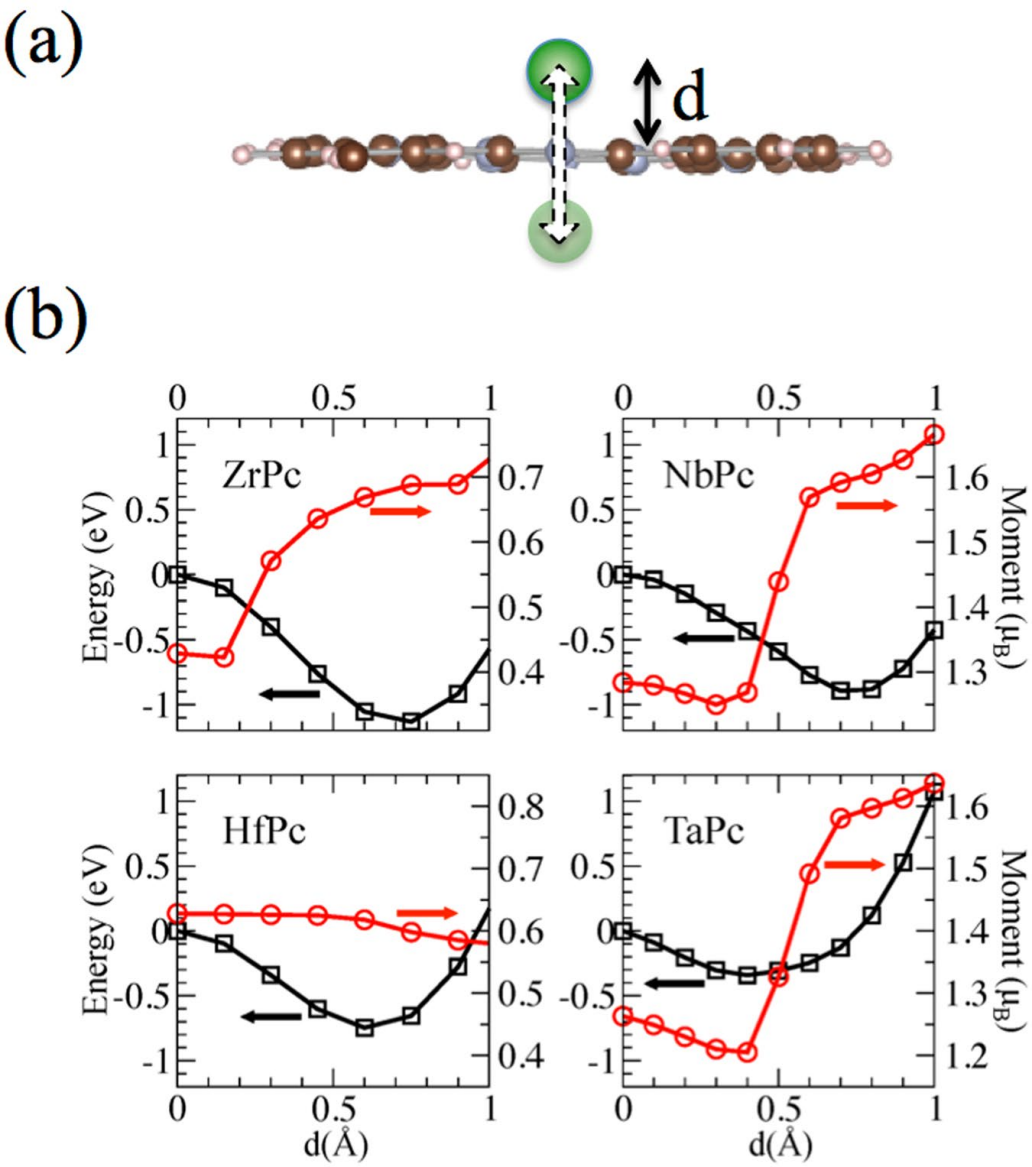
(**a**) Molecular structure used for the estimation of the energy barrier relative to the displacement of the metal ion from one side to the other of an isolated Pc macrocycle. The Pc macrocycle is flat and its atoms are held fixed. The metal ion is shifted rigidly along the molecule axis. (**b**) Energy profile (black square) for the transition described in (**a**) for ZrPc, NbPc, HfPc, and TaPc molecules. The red circles show the values of the magnetic moments of the metal ions (right axis).

We thus performed systematics calculations of MPc, where M is a transition metal ion belonging to the 4*d* and 5*d* series, rigidly shifting the M ion across the Pc plane (which has been here for simplicity assumed planar see Fig. 2(a)), allowing no relaxation of the Pc atomic coordinates. In this way we clearly overestimate the energy barrier that eventually exists between the two structural conformations so that elements that result in a planar geometry can be safely discarded. The ionic radius decreases monotonically along the 4*d* and 5*d* series due to incomplete screening of the nuclear charge. Thus, as the electron filling progresses, the spatial extent of the outer *d* shells recedes and eventually the ions fit into the Pc plane. We find that the cross-over between shuttle-cock and planar molecules occurs for five electrons in the 4*d* or 5*d* shell, i.e. for Tc, Re, and elements later in the series the structural energy minimum corresponds to the planar molecule. We therefore focus on the beginning of the series, where the *d* shell is less than half filled. In the case of Mo and W an energy barrier between the *up* and *down* conformations is present, however very low, namely of the order of 0.1–0.2 eV, and likely to disappear after adsorption of the molecule on a substrate. We are then left with the four elements of the IV and V groups, Zr, Nb, Hf, and Ta, on which we concentrate in the following.

### Structural, electronic and magnetic properties of free MPc molecules, with M = Zr, Nb, Hf and Ta

In Fig. 2(b) we plot the total energy and the magnetic moment on the TM ion as a function of the distance *d* between the TM and the Pc plane, with TM = Zr, Nb, Hf, and Ta. Total energy minimum is attained for *d* ≈ 0.75 Å for the 4*d* ions, while the 5*d* ions protrudes less as compared to 4*d*, i.e. *d*≈0.6 Å and 0.4 Å for Hf and Ta, respectively. The larger out-of-plane relaxation is reflected in the higher energy barriers of Zr (1.1 eV) and Nb (0.8 eV) as compared to the Hf (0.7 eV) and Ta (0.3 eV).

Concerning the magnetic properties, the elements of the IV group, Zr and Hf, have a valence electronic configuration ${d}^{2}{s}^{2}$ and, in our calculations, the balance between the electron affinities of the ions and of the Pc results in a +3 valency, with the donation of three electrons to the organic Pc ring, which however tends to accept only two excess electrons. This leads to a total spin S = 1 (i.e. M_s_ = 2*μ*_*B*_) in the molecule, arranged in an unpaired ${d}_{{z}^{2}}$ electron on the Zr or Hf ions, and a spin radical distributed over the Pc plane. Elements of the V group, i.e. Nb and Ta, have an electron more in the valence, but a similar tendency towards the +3 oxidation state, which results in 2 valence electrons. The interplay between crystal field splittings and Coulomb repulsion energy induces a high-spin state, accommodating the second electron into one of the higher-energy degenerate ${d}_{xz+yz}$ level, and leading thus to a S = 3/2 molecular spin.

The TM magnetic moments plotted in Fig. 2(b) are smaller than the nominal ones since they are derived from an integration of the spin density over a confined sphere around the nuclei, whereas the fraction of electrons spilling out from these spheres is lost. Moreover, for all the metal ions except Hf, the magnetic moment localized on the TM ion decreases by about 0.3–0.4 *μ*_*B*_ upon shifting towards the center of the Pc plane, accompanied by a charge redistribution from the delocalized Pc *π* orbital to the ion. If structural relaxation of the atomic coordinates is carried out, the Pc plane bends away from the metal ion for electrostatic reasons similarly to SnPc and evidenced in Fig. 1(b), whereas only minor modifications in the local magnetic moment on the metal ion are observed.

### Characterization of MPc molecules on graphene decorated Ni(111) substrate

When non-planar metalorganic magnetic molecules are adsorbed on a substrate, several scenarios can occur depending on the strength of the interaction. Electron transfer from or towards the molecule could result in detrimental effects on the structure, e.g. loss of the non-planar geometry or instability of the *up*-configuration, or on the magnetic properties, e.g. quenching the magnetic moment. The choice of the substrate is thus crucial when designing a molecular spintronics device, in which specific functionalities that molecules have in the gas phase want to be utilized.

We first considered a metallic surface that is widely employed for conducting devices, namely Cu(111) and found that due to the large reactivity of the substrate, no stable *up* conformation for the MPc molecules is present, i.e. the barrier is flattened out (see Supplementary Figure S1). The molecule-surface interaction is known to be reduced by the presence of a spacer organic or insulating layer^35, 36^. Specifically, graphene (G) has been shown to act as an electronic decoupling layer able to preserve to a large extent the properties of adsorbed molecules and atoms, and simultaneously allow magnetic communication between the adsorbate and the substrate magnetization^27–31, 37^. We then adopted the graphene decorated Ni(111) substrate (G/Ni(111)), a system where the graphene layer adsorbs pseudomorphically.

We have characterized the four MPc molecules discussed in Fig. 2 adsorbed on a top-fcc-stacked graphene layer on the Ni(111) surface (the Ni slab has been modeled with four Ni monolayers); structural relaxations have been performed for the molecule, graphene and topmost Ni layer atoms, until remanent forces were smaller than 0.02 eV Å^−1^. We observe that for the V group elements, Nb and Ta, whenever structural relaxations are initiated, although different *up* initial positions are tried, the system falls into the *down* state, meaning that the interaction with the metal substrate, although reduced, is strong enough to flatten out the energy barrier, i.e. only the *down* state is stable.

Instead, in the case of the IV group ions, i.e. Zr and Hf, both *up* and *down* structural states are stable after atomic relaxation. We estimated the energy barrier for the transition between the *up*- and *down*-configurations for the ZrPc and HfPc molecules on G/Ni(111) based on the nudge elastic band method (NEB)^38^, for which we adopted the fully-relaxed molecule-surface geometrical structures of the *up*- and *down*-configurations as starting and final images. This method accounts for structural relaxation of the molecules also in the intermediate, out-of-equilibrium configurations to ensure the minimum energy path is found.

**Figure 3:**
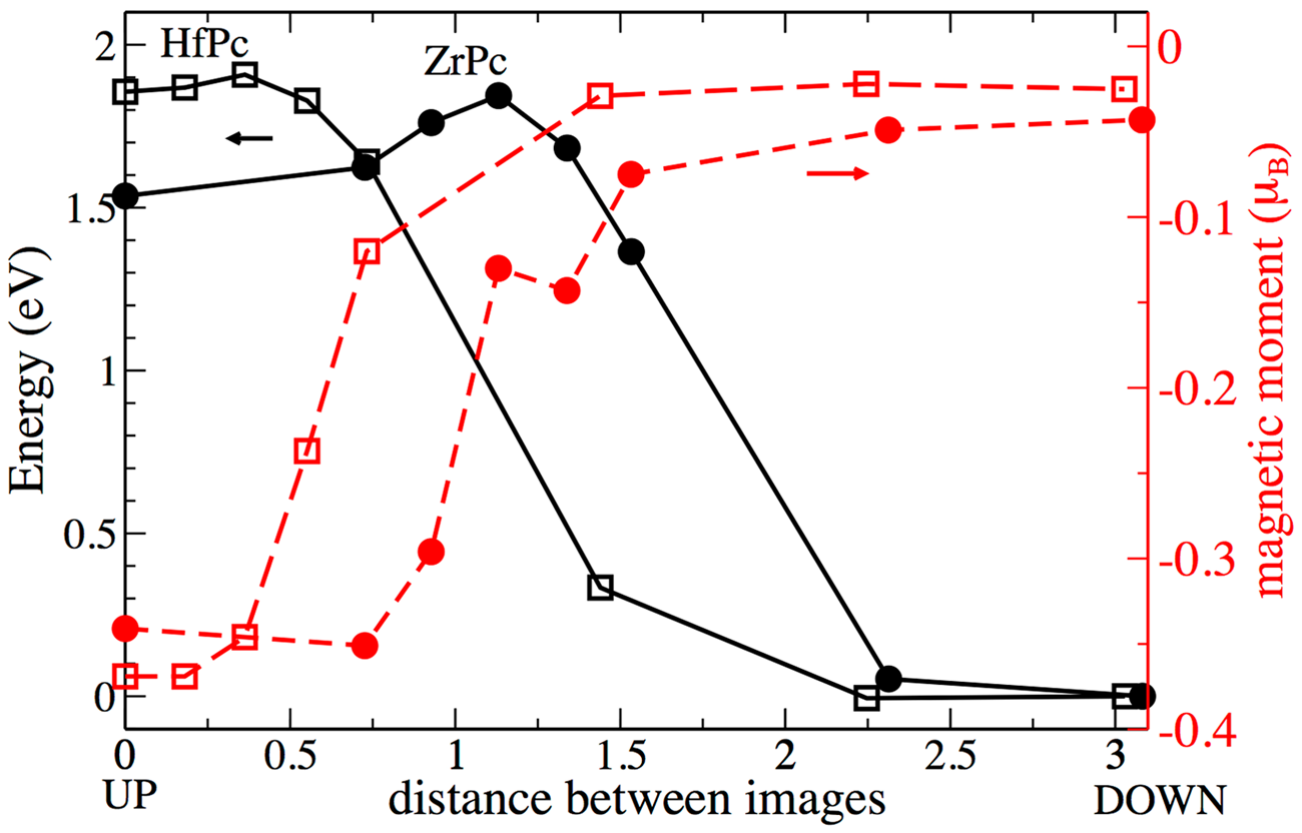
Energy barrier (black solid line) relative to the transition between the *up*- and *down*-configurations for HfPc (empty symbols) and ZrPc (full symbols) molecules on G/Ni(111) evaluated based on the NEB method. The magnetic moment of the Zr and Hf atoms are plotted with red dashed lines and referenced to the right axis. The distance between the images is defined as the root-mean-squared distances between all atoms.

In Fig. 3 the energy barrier profile obtained by the NEB method is shown together with the magnetic moments on the metal ions. The energy barrier is different depending on the transition direction: in the *up*-to-*down* transition it is about 0.1 eV and 0.3 eV for the ZrPc and HfPc molecules, respectively, while in the *down*-to-*up* transition is larger, around 1.8–1.9 eV for both molecules, which is a consequence of the stronger bond of the Zr and Hf ions with the surface in the *down* configuration. During the *up*-to-*down* transition, the magnetic moment of the metal ions is gradually reduced, starting from values slightly lower than 0.4, *μ*_*B*_ and becoming essentially quenched in the *down* configuration (see Supplementary Figure S2).

**Figure 4:**
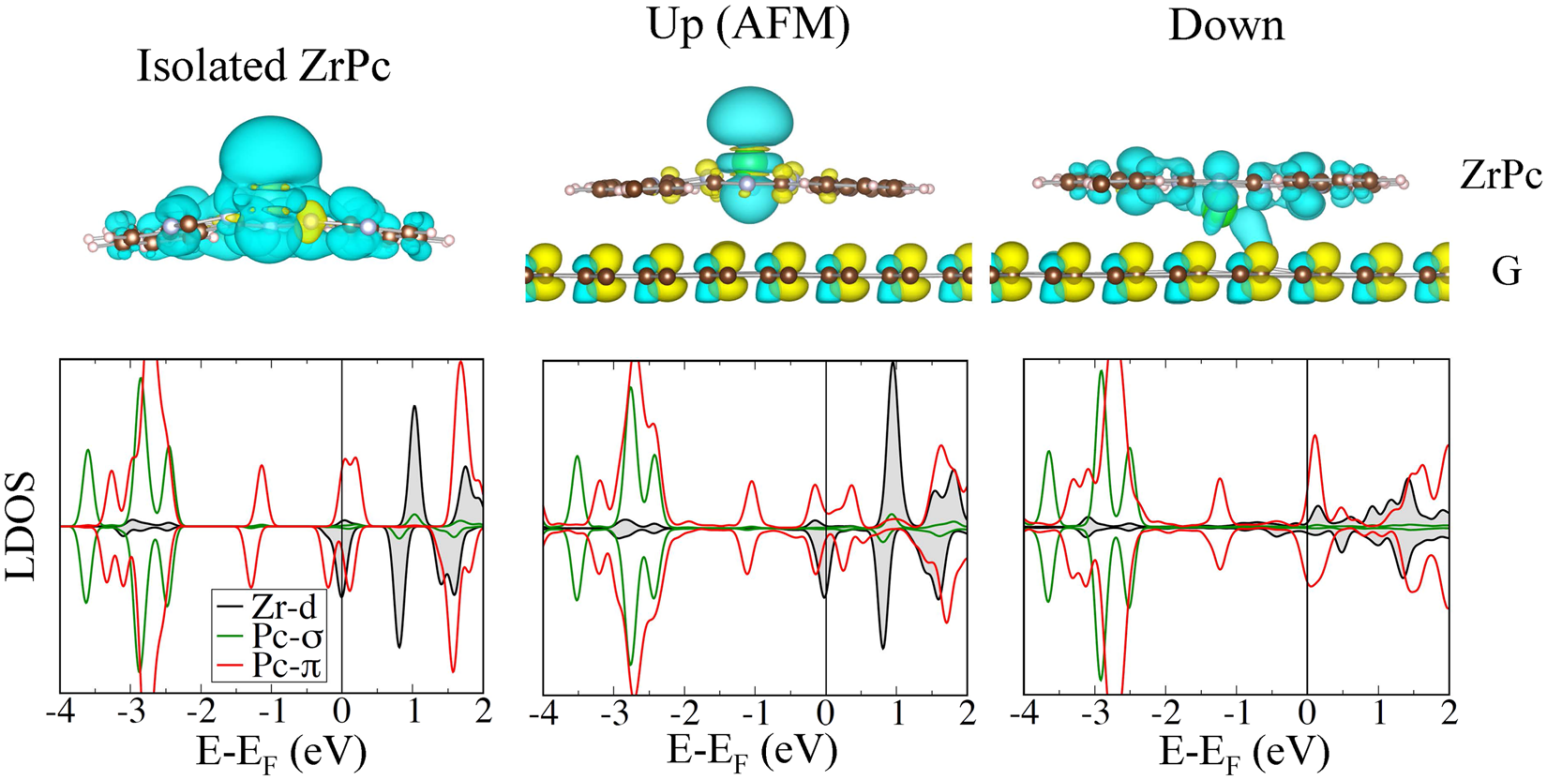
Plots of the spin densities (top panels) and of the spin-resolved LDOS (bottom panels) for an isolated ZrPc molecule (left) and for a ZrPc molecule on G/Ni(111) in the *up*- (center) and *down*-configuration (right). For the *up*-configuration, the plots refer to the (ground state) case of antiparallel alignment of the Zr and Ni magnetic moments (the spin density as well as LDOS of the Ni substrate is not depicted in the plots, for clarity reasons). Cyan (yellow) corresponds to positive (negative) spin density (see text for further details).

In order to comprehend what happens to the electronic states of the entire molecule, it is instructive to compare the magnetization density and the local density of states (LDOS) of the gas-phase molecule, and of the molecule in both the *up* and *down* conformations on the G/Ni(111) substrate. The discussion is presented in Fig. 4 only for ZrPc, for simplicity, but a similar scenario occurs also for HfPc. For an isolated ZrPc molecule, the magnetization is carried by the spin-polarized HOMO which contains a *d* state of the Zr ion and a *π* state of the Pc-macrocycle; in Fig. 4, top-left panel, an excess number of majority (minority) electrons leads to a positive/yellow color (negative/cyan color) spin density. Here, the case of a negative molecular spin is considered to better compare with the LDOS and spin density of the molecule-substrate systems discussed below. When adsorbed on G/Ni(111) in the *up* configuration, the polarization on the Pc (radical) vanishes due to the interaction with the substrate, whereas the electronic structure of the metal center (far from the graphene layer) is essentially unaffected, although a reduction in the integrated spin is observed. The spin density on the Zr ion is reduced and is negative (cyan color), since antiparallel coupling with the Ni magnetization is obtained in the ground state. Looking at the LDOS (bottom-left and -center panels), the fact that modifications are limited upon absorption in the *up* state indicates a physisorption mechanism. In the *down* state, the hybridization with the substrate broadens the *d* states of Zr, which is now chemically bound to graphene, resulting in the almost complete loss of its magnetic moment (bottom-right panel). The quenching of the transition metal ion magnetic moment from the *up* to *down* state is related to an intramolecular redistribution of the spin polarization from a ${d}_{{z}^{2}}$ state localized on the metal center to a delocalized *π* orbital, i.e. the molecules in the *down* state retains a magnetization that is distributed on the Pc macrocycle (top right panel). In fact, although the overall LDOS of the Pc organic cycle is not much modified in the *down* configuration, close to the Fermi level a net negative polarization is induced on the HOMO *π* orbital of the Pc ring indirectly via the Zr ion, as shown in the bottom-right panel of Fig. 4. Moreover the adsorption of the molecules is found to induce very small modification in the states of the graphene layer, where the well known alternating spin polarization of the two C atoms (top and fcc stacked on Ni) is observed. In Supplementary Figure S2, the evolution of the spin density moving from the *up* to *down* states of a ZrPc molecule on G/Ni(111) is depicted also for intermediate NEB images, i.e. when the Zr ion is passing through the Pc plane.

The spin moment of Zr and Hf in the *up* state is intrinsic and could be converged either parallel or antiparallel to the Ni moments. By comparing the total energies of the different solutions we observe that the antiparallel state is more stable than the parallel one by around 35 meV and 25 meV, for ZrPc and HfPc respectively. Instead, the polarization of the Pc in the *down* state is extrinsic and only caused by contact interaction with the substrate, i.e. its sign could not be set independently with respect to the Ni.

The value of the antiferromagnetic coupling observed in the *up* state is rather large, and should be sufficient to stabilize the molecule’s magnetic moment against thermal fluctuations, allowing its measurements in standard low temperature XMCD experiments, even in the absence of anisotropy-induced magnetic bistability (as in molecular magnets). The fact that the size of the interaction amounts to several tens of meV despite the presence of graphene decoupling layer is not surprising, and has a twofold origin. We have already demonstrated in previous work that despite the electronic decoupling character of the graphene layer, its ability to spin-polarize its states is the key to mediate efficiently the magnetic coupling through it. For instance in case of a FePc directly adsorbed on Ni(111), the (ferromagnetic) coupling has been found to amount to 73 meV being reduced to 14 meV (remaining ferromagnetic) in presence of the graphene decoupling layer^29^. In that case the spin moment is localized in the 3d orbital of Fe ion, which are less spatially extended compared to the 4d (5d) orbitals of Zr (Hf) ions. In addition, in the current work, the presence of the spin radical induces a larger electron transfer between the molecule and the substrate, toughening the magnetic coupling.

Moreover, our observation of an antiferromagnetic coupling is consistent with the fact that, in the isolated molecule, both the unpaired 4d(5d) electron as well as the spin radical in the Pc plane are hosted in electronic states pinned at the Fermi level. These states overlap (in space and in energy) with the electronic states of the substrate, which is also characterized by having states close to the Fermi level with predominant minority spin channel character (not shown), supposing a positive Ni magnetization. Therefore energy matching between the molecule and the substrate requires the spin of the molecule to be opposite to the one of Ni. The same mechanism has been found to explain the antiferromagnetic coupling between a cobaltocene spin and Ni as well G/Ni(111) substrates, as discussed in a previous paper from our group^27^.

If on one hand the coupling with the Ni magnetization should stabilize the magnetic moment of the molecules, on the other 4d and 5d transition metals are known to present larger relativistic effects in their electronic shells. We attempted to extract informations on the preferred orientation of the magnetization axis of the molecules, by including spin-orbit coupling in the calculations. In pseudopotential codes such as VASP (or Quantum Espresso) spin-orbit is implemented within a non-collinear framework, and this type of calculations require as much as 8-10 times more computational resources, because of the spinorial formulation of the wave functions. In addition, for extended system such as a Ni surface, usually a large number of k-points are necessary in order to obtain reliable information on the magnetic anisotropy energy (MAE). Thus, the extraction of the MAE and the determination of the easy axis of magnetization would be a formidable task for the molecule-substrate systems, and we limited ourselves to calculate the MAE for the isolated ZrPc and HfPc molecules. We observe for ZrPc a preference to orient the magnetization along the axis perpendicular to the Pc plane, i.e. easy axis anisotropy, MAE = 2 meV, while for HfPc an easy plane anisotropy has been found, with a MAE of 1 meV. The net effect on the structural relaxation as well as charge transfer upon adsorption on the G/Ni(111) substrate could not be foreseen, and could easily modify the behavior shown by the isolated molecules.

**Figure 5:**
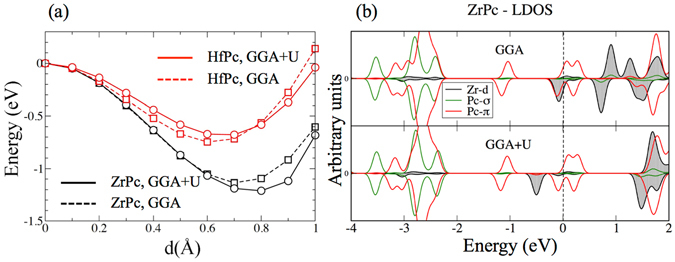
(**a**) Comparison between the energy barriers relative to the transition between the *up*- and *down*-configurations for ZrPc (black lines) and HfPc (red lines) molecules, calculated with the same method used in Fig. 2(b), where continuous (dashed) lines - circle (square) symbols refer to the GGA + U (GGA) calculations. d is the distance between the Zr(Hf) ion and the Pc plane. (**b**) Comparison between the spin-resolved LDOS for an isolated ZrPc molecule for the GGA (upper panel) and GGA+U (lower panel) methods.

Finally, although there is no clear consensus in the literature if correlation effects play a role in 4d and 5d orbitals, being more extended in space with respect to the 3d ones, and considering that they are more relevant in the middle/second half of the d series, where more electrons share the same region of space, we have included them by means of the LDA + U method (see Method section for more details), in order to test the robustness of our results. We observe that upon inclusion of correlation effects: (i) The *up* conformation remains stable and the Zr (Hf) ions attained, upon re-optimization of the atomic coordinates, similar out-of-plane displacement from the Pc group, a clear sign that the size of the barrier is similar to the one obtained within the GGA approximation. This is evidenced in Fig. 5(a), where the comparison between the energy barriers for ZrPc (black lines) and HfPc (red lines) isolated molecules, calculated with the same method used in Fig. 2(b), with (continuous lines) and without (dashed lines) correlation effects is presented. (ii) The difference in total energies between the parallel and antiparallel coupling in the *up* state is slightly modified, becoming 16 meV for ZrPc and and 32 meV for HfPc, but the preference for the antiferromagnetic coupling is preserved, and very small changes are observed in terms of the residual magnetic moments in the molecule (see Supplementary Figure S3). (iii) Except a scissor-like increase in the energy distance between the occupied and unoccupied d orbitals, the electronic states of the molecule are barely affected, as shown for ZrPc as an isolated molecule (in Fig. 5(b)) and adsorbed on the G/Ni(111) substrate (see Supplementary Figure S4). This is due to the fact that in the ZrPc and HfPc molecules the unbalance between the electron affinities of the 4d(5d) ion and the organic Pc leads to an unpaired electron in the *π* Pc orbital that juxtaposes with the unpaired electron in the central ion d orbital. The electronic states of this organic radical are pinned at the’Fermi level’ or more precisely hosted in a singly occupied molecular orbital (SOMO), delocalized over the Pc. The inclusion of correlation effects in the 4d or 5d orbital therefore has basically no effect on the energy position of the spin radical, occurrence that prevents the opening of a HOMO-LUMO gap in the molecule in a DFT calculation.

### Comparison with SnPc and PbPc molecules

It is instructive to compare to the case of the SnPc and PbPc molecules on Ag substrate discussed in ref. 26 Experimentally, when the SnPc molecule is in direct contact with the Ag substrate, irreversible *up*-to-*down* switching is observed, the energy necessary to induce it being around 1.5 eV. When the same conformation transition is undertaken for a SnPc that is instead adsorbed on a buffer SnPc layer, the barrier is increased to 2.5 eV. The fact that the *down*-to-*up* transition is not possible when the SnPc is in direct contact with Ag implies that the energy barrier is larger than the highest voltage that it is possible to apply without detrimental effects for the molecule. Moreover, in the case of SnPc on a SnPc layer, the barrier felt in the *down*-to-*up* transition is larger than in the *up*-to-*down* case, as evidenced by the larger persistence in the *down* state in Fig. 4(b) of ref. 26 This behavior is compatible with a barrier profile similar to the one we observe for ZrPc on G/Ni(111) shown in Fig. 3. In the case of PbPc, where the shuttlecock shape is also attained, the barrier felt by the Pb ion to cross the Pc plane is too large, thus no switching is observed^39^. The interaction with the substrate is sought on purpose in order to lower the energy barrier between the two conformations, which would otherwise be too large and prohibit the switching. As discussed above, despite the presence of the graphene decoupling layer, the *down* state is more stable than the *up* state, and it is required a larger energy to flip from the *down* to the *up* state than vice versa. Nevertheless, all the energy barriers are smaller than for SnPc molecules on a SnPc layer on Ag(111) and well within the experimental applicable voltages. We note that a feasible way to favor a reversible change in the magnetic state, could be supplied by molecule functionalization, adding for instance, bulky peripheral ligand, increasing the adsorption distance between the molecule and the substrate and thus decreasing the barrier present during the *down* to *up* switching event.

**Figure 6:**
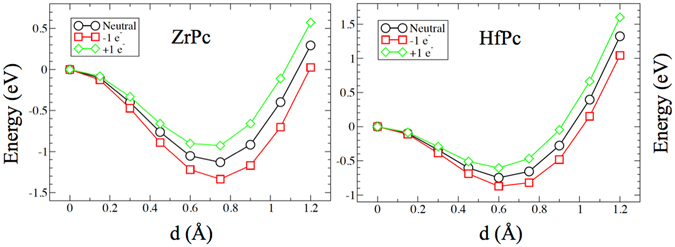
Energy barriers relative to the transition between the *up*- and *down*-configurations for ZrPc (right panel) and HfPc (left panel) molecules, calculated with the same method used in Fig. 2(b). d is the distance between the Zr(Hf) ion and the Pc plane. The calculations have been carried out for the neutral molecules (black lines), in the presence of an additional hole (red lines) and an additional electron (green lines) in the cell.

Dynamical effects that appear when an electronic current flows through a molecule are difficult to be taken into account in standard DFT calculations. One would in principle need to employ time-dependent DFT, or at least molecular-dynamic calculations, in order to account for vibrational degrees of freedom and excited states. Instead, in the following, we present an approximated method to simulate the transient state of a tunneling process, simulating the instantaneous charging experienced by the molecule when crossed by an STM tunneling current, as already proposed in the original paper of Wang *et al*. on the SnPc molecule^26^. We found a small but sizable influence of charging which is of the order of ≈0.2 eV on the energy barrier, as evidenced in Fig. 6, which suggests that current or voltage induced charging of the molecule can induce the *up*-to-*down* structural transition.

We note that phthalocyanines and porphyrins containing metal ions of the IV group, i.e. Zr and Hf, have been synthesized so far in the presence of additional central ligand atoms, e.g. Cl^−^ atoms, due to the preferred 4+, and less abundant 3+, oxidation states of Zr and Hf. Further reduction could be achieved by surface chemistry, by, stimulated detaching of the Cl ions. Lower oxidation states have also been achieved by synthetic chemistry^40–42^. Alternatively, atomic manipulation/metalation could also be a viable method to construct such Pcs directly on the surface, starting from non metal H_2_ Pc derivatives, and exploiting the capability of STM tips to pick and deposit single atoms to construct atomic aggregates with tailored geometries^43^.

## Conclusions

To summarize, we have undertaken a proof-of-concept quest to find a magnetic metalorganic molecular system with two different magnetic state, whose magnetic and structural properties are strongly interrelated – switching between two structural states results in magnetic moment quenching or creation. We find ZrPc and HfPc molecules to be good candidates to implement a magnetic *molecular button* behavior, when deposited on a low interacting G/Ni(111) substrate. Due to the large exchange interaction with the Ni(111) substrate, the magnetic moment is stable at low temperature and the two different states can be distinguished/sensed also by measurements sensitive only to the magnetic structure. We have also shown that the interaction between the molecular spin and the substrate leads to a spin polarization in the molecule, when the Zr (Hf) ion is below the Pc plane, which is otherwise not present for the isolated molecule, a results which contextualizes itself in the more and more investigated field of spinterface physics. We believe that this work will stimulate the interest of experimentalists, together with further theoretical efforts towards the creation of novel molecular magnetic switches with tailored properties.

## Methods

The DFT calculations were carried out using the Vienna ab-initio simulation package (VASP)^32, 33^ where GGA-PAW pseudopotentials were employed, together with empirical van der Waals dispersion terms^44^. A cut-off energy of 450 eV for the plane wave basis set and a kinetic energy cut-off of 900 eV for the augmentation charges have been used. We simulated graphene on Ni(111) using a 4-ML-thick Ni slab with one graphene layer adsorbed on one side. About 10 Å of vacuum space is inserted to avoid spurious interactions between the molecule and the slab replicas. A (8 × 8) in-plane unit cell containing 128 C and 64 Ni atoms/layer (for a total of 441 atoms, considering also the MPc molecule, in the simulation cell) and a 2 × 2 k-points grid have been employed for the calculations of the electronic and magnetic properties. Structural relaxations have been performed for the M-Pc molecule, the graphene, and the topmost Ni layer (Γ-point calculation), until remanent forces were smaller than 0.02 eV/Å, while the other Ni atoms were held fixed in the equilibrium position of the bare Ni(111) surface. Static correlation effects have been included (when explicitly stated) including Hubbard-type +U corrections as implemented by Liechtenstein *et al*.^45^, to verify the robustness of the GGA results. An on-site potential term of U = 4 eV (and J = 0 eV) has been considered acting on the 4d and 5d orbital. Upon inclusion of correlation effects, the atomic coordinates both for the isolated molecules and for the molecule-substrate systems have been re-optimized. In order to extract the magnetic anisotropy energy (MAE) and easy axis of magnetization of the isolated ZrPc and HfPc molecules, non-collinear calculations including spin-orbit coupling have been carried out, performing at first a collinear calculation from which the charge density, as well as the atomic coordinates, are read in and kept frozen during the self-consistency cycle. The MAE is obtained as the difference between the total energy obtained placing the magnetization direction perpendicular to the Pc plane (the z axis of the simulation cell) and an average of two total energies obtained setting the magnetization axis along two (perpendicular to each other) axis in the Pc plane (the x and y axes of the simulation cell). A negative MAE indicates an easy(z)-axis magnetization, while a positive MAE indicates an easy(xy)-plane magnetization.

## Electronic supplementary material

Supplementary Info
