# Supplementary material for: Designing a molecular magnetic button based on 4d and 5d transition-metal phthalocyanines
Source: Sci Rep. 2017 Jun 16;7:3647. doi: 10.1038/s41598-017-03920-5 (PMC5473879; doi:10.1038/s41598-017-03920-5)
Supplement: Supplementary file 1 — Supplementary Info [file 41598_2017_3920_MOESM1_ESM.pdf]

Designing a molecular magnetic button based on $4d$ and $5d$ transition-metal phthalocyanines:

Supplementary information

P. Ferriani [1], S. Heinze[1] and V. Bellini[2,†]

[1] Institute of Theoretical Physics and Astrophysics, University of Kiel, D-24098 Kiel, Germany.
[2] S3-Istituto di Nanoscienze-CNR, Via Campi 213/A, I-41125 Modena, Italy.
[†] valerio.bellini@unimore.it

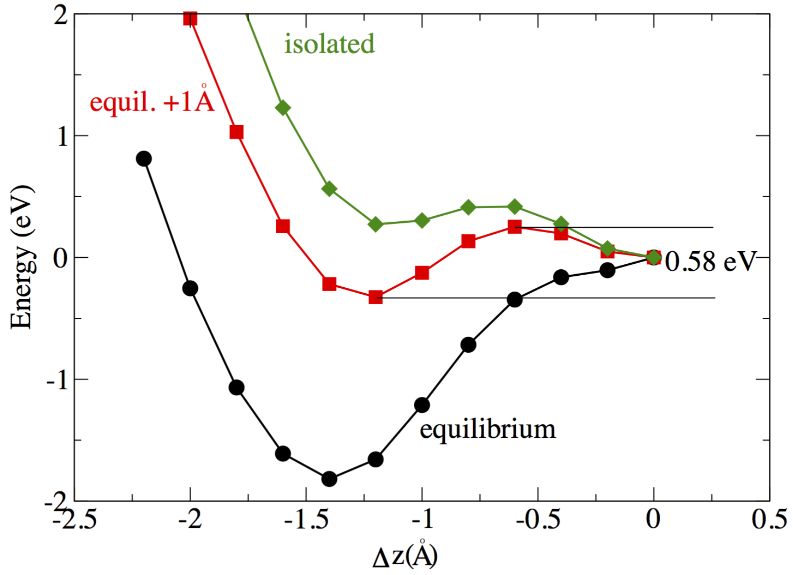

Supplementary Figure S1: Interaction between NbPc molecule and Cu(111) substrate. We tested how the energy barrier between the two shuttlecock stable conformations *up* and *down* depicted in Fig.1(c) (main text) is influenced by the interaction with a reactive surface, placing a NbPc molecule at different distances from a Cu(111) surface. The black curve is relative to the equilibrium distance (total energy minimum obtained varying rigidly the molecule surface distance), the red curve is obtained increasing this distance by 1 Å , and the green curve is relative to a molecule far away from the surface (isolated molecule). Each point in these curves represents a calculation where only the Nb ion is shifted along the 111 (z) direction perpendicular to the surface/Pc plane by a quantity Δz (the origin in the abscissas axis is when the Nb ion is in the original position of the *up* conformation), while the total energy of each calculation is reported in the coordinate axis (the zero is the total energy of a molecule in the *up* conformation at equilibrium distance from the Cu surface).

The energy barrier which stabilizes the *up* conformation is completely flattened out when the molecule is at equilibrium distance from the Cu surface (black curve); in fact no minimum is found for $\Delta z = 0$ while a deep energy minimum is observed when the Nb ions is shifted towards the surface and below the Pc plane by $\Delta z = -1.4$ Å(close to where it would be in the *down* conformation). Upon shifting the whole molecule by 1 Å (red curve), the interaction with the surface is reduced and the energy barrier which stabilizes the *up* conformation is regained, although reduced by half, as compared to the barrier felt by the Nb ion when crossing the Pc plane from below, i.e. 0.28 eV *cf.* 0.58 eV. The curve for the isolated molecule is inserted for comparison (green curve); note that the Nb position in the *down* conformation would at $\simeq \Delta z = -1.2$ Å and the curve is not symmetric around -0.6 Å , because in this calculation the Pc plane retains the curvature of the *up* conformation when the Nb ion is rigidly shifted by Δz, differently from the ones discussed in Fig. 2(b) in the main text, where the Pc plane is assumed flat.

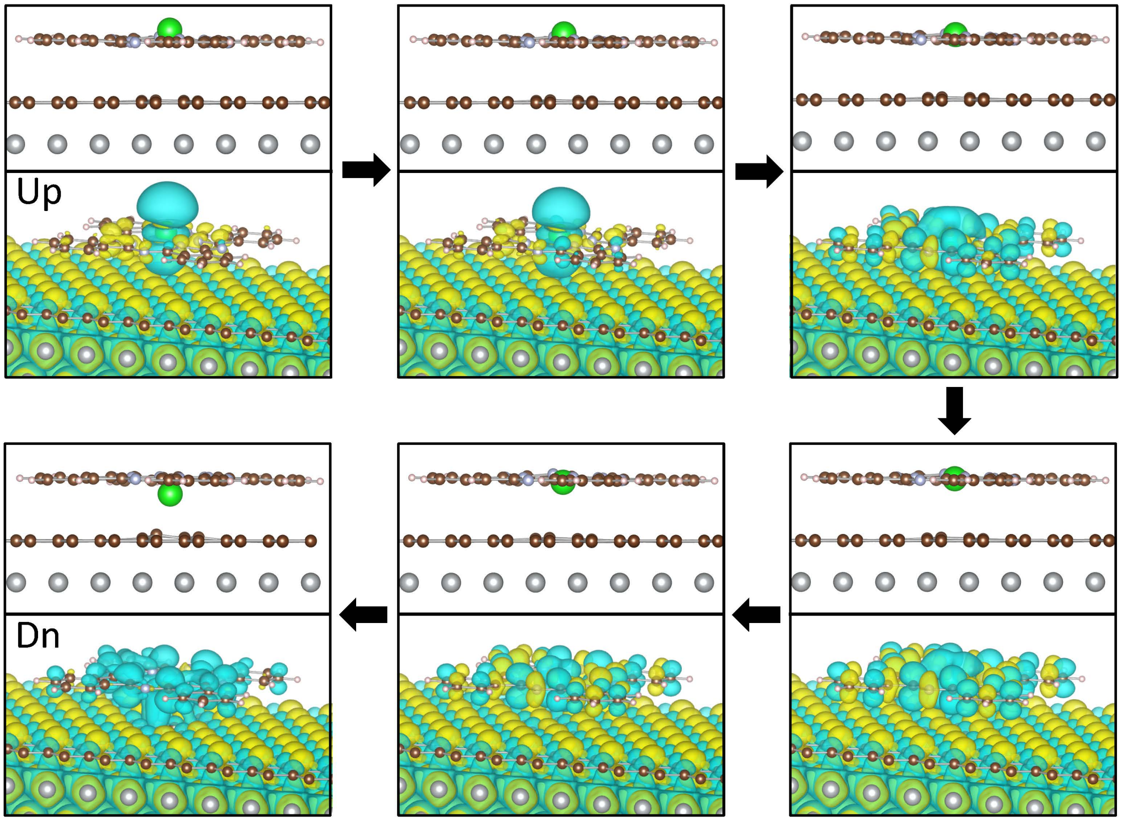

Supplementary Figure S2: Spin density analysis for ZrPc on G/Ni(111) for Zr-Ni antiparallel magnetic coupling. The spin densities are depicted for different intermediate NEB states between the two extrema, i.e. the *up* and (b) the *down* configurations shown in the upper and lower left panels, in order to visualize the progressive loss of the magnetic moment on the Zr ion and the consequent modifications on the Pc polarization which follows the increase in the charge transfer between the molecule and the G/Ni surface, observed when moving from the *up* to the *down* configurations. Cyan (yellow) color indicates an excess of spin down (up) electrons. The spin polarization at the Zr ion is already reduced when the ion is within the Pc plane, and at this stage a clear modification of the spin-polarization of the Pc plane is also observed (both colors are present). When the *down* conformation is reached the negative spin polarization on the Zr ion is barely visible, while a clear negative spin polarization pops up throughout the Pc plane.

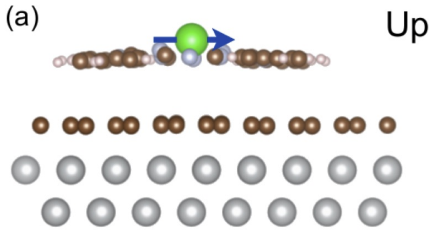

Zr Pc	M_{Zr} (μ_B)		E_{AFM-FM} (meV)	
	Up	Dn	Up	Dn
U-J=0	-0.34	0	-34	0
U-J=4	-0.58	0	-16	0

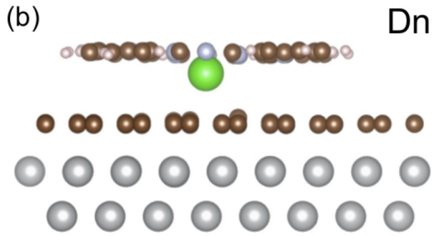

Hf Pc	M_{Hf} (μ_B)		E_{AFM-FM} (meV)	
	Up	Dn	Up	Dn
U-J=0	-0.37	0	-25	0
U-J=4	-0.55	0	-34	0

Supplementary Figure S3: magnetic properties of ZrPc ad HfPc molecules on G/Ni(111) with GGA and GGA+U functionals. In panel (a) the *up* and (b) the *down* configurations after structural relaxation are depicted for a ZrPc molecule adsorbed on G/Ni(111). The Zr ion is represented in green. C, N, H, Ni atoms are colored brown, light grey, white, dark grey, respectively. The structure of a HfPc molecule is very similar and is not shown. On the right, the tables summarize the magnetic moments (M) of the Zr and Hf ions for each adsorption configuration evaluated in the GGA and GGA+U schemes. The magnetic moment of the Zr and Hf ions is quenched in the *down* configuration when the metal center is beneath the Pc plane and close to Ni. The energy difference (E_{AFM-FM}) between the configurations with the molecular moment antiparallel and parallel to the Ni moment, respectively, which stems from the molecule-substrate exchange interaction is also listed for the *up* structural configuration.

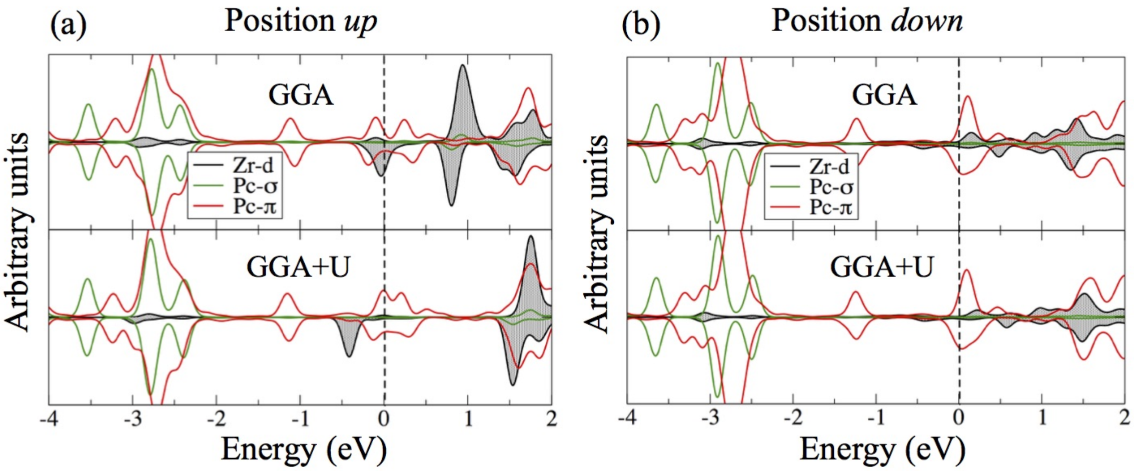

Supplementary Figure S4: Comparison between the spin-resolved LDOS calculated by the GGA and GGA+U methods for the ZrPc molecule on G/Ni(111) in the (a) *up* and (b) *down* structural conformations. Static correlation effects induce a scissor-operator like increase in the energy distance between the occupied and unoccupied d orbitals, but the electronic states of the molecule are barely affected. The electronic states of the organic radical in the *up* configuration are hosted in a singly occupied molecular orbital (SOMO), delocalized over the Pc, which are not modified by the inclusion of correlation effects in the 4d or 5d orbital.
